# Supplementary material for: MetaRibo-Seq measures translation in microbiomes
Source: Nat Commun. 2020 Jun 29;11:3268. doi: 10.1038/s41467-020-17081-z (PMC7324362; doi:10.1038/s41467-020-17081-z)
Supplement: Supplementary file 10 — Supplementary Data 7 [file 41467_2020_17081_MOESM10_ESM.zip › File2/Confidence_High_Taxonomy/298737_out.krona.html]

Javascript must be enabled to view this page.

members
magnitude
magnitudeUnassigned
count
unassigned
taxon
rank

298737\_out

38

38
2
superkingdom

1239
phylum
38

186801
class
2

2
order
186802

1
541000
family

1
216851
genus


SRS142542\_contig\_number\_contig-100\_1490.1491
1
853
species

1
216572
family

1
459786
genus

1

SRS097889\_contig\_number\_16389
species
876091

36

SRS051610\_contig\_number\_contig-100\_26085.26086SRS051882\_contig\_number\_contig-100\_398.93618SRS075078\_contig\_number\_17825SRS076756\_contig\_number\_7705SRS076976\_contig\_number\_contig-100\_153.44657SRS077194\_contig\_number\_8617SRS077392\_contig\_number\_contig-100\_16634.16635SRS077454\_contig\_number\_contig-100\_7216.7217SRS077502\_contig\_number\_2811SRS097920\_contig\_number\_2904SRS097958\_contig\_number\_contig-100\_11470.11471SRS098571\_contig\_number\_35021SRS098655\_contig\_number\_contig-100\_36768.36769SRS098717\_contig\_number\_9244SRS1041136\_contig\_number\_14748SRS1041138\_contig\_number\_2086SRS104327\_contig\_number\_1304SRS104912\_contig\_number\_6758SRS1055043\_contig\_number\_375SRS140492\_contig\_number\_13333SRS143085\_contig\_number\_contig-100\_2828.44951SRS143148\_contig\_number\_1444SRS143466\_contig\_number\_7307SRS143523\_contig\_number\_21128SRS143722\_contig\_number\_contig-100\_2053.35490SRS143780\_contig\_number\_contig-100\_14507.139883SRS144362\_contig\_number\_contig-100\_4945.230281SRS144506\_contig\_number\_22596SRS146764\_contig\_number\_7295SRS147377\_contig\_number\_278SRS148253\_contig\_number\_5169SRS148721\_contig\_number\_41598SRS148784\_contig\_number\_13086SRS149075\_contig\_number\_6660SRS149879\_contig\_number\_contig-100\_766.50681SRS893358\_contig\_number\_contig-100\_16404.16405
species
1262992
